# Cathepsin K inhibitors increase distal femoral bone mineral density in rapidly growing rabbits

**DOI:** 10.1186/1471-2474-14-344

**Published:** 2013-12-09

**Authors:** Brenda L Pennypacker, Renata M Oballa, Sonia Levesque, Donald B Kimmel, Le T Duong

**Affiliations:** 1 Inception Sciences Canada Inc., Suite 210, 887 Great Northern Way, V5T 4T5, Vancouver, BC Canada; 2 AniDis, 4850 chemin Bois Franc, Suite 200, St-Laurent, H4S 1A7, Province of Quebec, Canada; 3 Osteoporosis Research Center, Creighton University, 601 North 30th Street, Omaha, NE 68131, USA; 4 Merck Research Laboratories, Merck & Co., Inc., P.O. Box 100, Whitehouse Station, NJ 08889, USA

**Keywords:** Odanacatib, Cathepsin K inhibitor, Osteoclast, Antiresorptives, DXA, Rabbit Schenk assay, Postmenopausal osteoporosis

## Abstract

**Background:**

Selective and reversible inhibitors of human Cathepsin K (CatK), including odanacatib (ODN), have been developed as potential therapeutics for the treatment of osteoporosis. Inhibitors of human CatK show significantly less potency for the rodent enzymes compared with that for the human or rabbit enzymes; thus the Schenk model in growing rabbit was developed as a screening assay for the *in vivo* activity of CatK inhibitors in blocking bone resorption.

**Methods:**

In this study, the efficacy of the selective inhibitors L-833905, L-006235, L-873724, and L-1037536 (ODN) of human CatK in the rapidly growing rabbit ‘Schenk’ model (age seven weeks) was compared to vehicle, using the bisphosphonate, alendronate (ALN), as a positive control, to assess inhibition of bone resorption. An enzyme inhibition assay (EIA) and an *in vitro* bone resorption assay using rabbit osteoclasts on bovine cortical bone slices were performed to evaluate the potency of these CatK inhibitors. Bone mineral density of the distal femur (DFBMD) was measured after ten days of treatment using *ex vivo* DXA densitometry.

**Results:**

Results of the EIA using rabbit CatK and the rabbit bone resorption assay showed that three of the four compounds (L-006235, L-873724, and ODN) had similar potencies in the reduction of collagen degradation. L-833905 appeared to be a weaker inhibitor of CatK. Taking into account the respective *in vitro* potencies and pharmacokinetic profiles via oral administration, the efficacy of these four CatK inhibitors was demonstrated in a dose-related manner in the growing rabbit. Significant increases in DFBMD in animals dosed with the CatK inhibitors compared to vehicle were seen.

**Conclusions:**

Efficacy of the CatK inhibitors in the Schenk rabbit correlated well with that in the *in vitro* rabbit bone resorption assay and in the ovariectomized rabbit model as previously published. Hence, these studies validated the rabbit Schenk assay as a rapid and reliable *in vivo* model for prioritizing human CatK inhibitors as potential therapeutic agents.

## Background

The lysosomal cysteine protease, Cathepsin K (CatK), is predominantly expressed in osteoclasts [1] and has an important role in the degradation of the demineralized collagen matrix components of bone (predominantly Type-I collagen) at neutral and acidic pH [2]. Genetic confirmation for a role of CatK in bone resorption exists in the form of pycnodysostosis, a rare human genetic disease linked to several loss-of-function mutations in the CatK gene characterized by high bone mineral density (BMD), acroosteolysis of the distal phalanges, short stature, and skull deformities with delayed suture closure [3-5]. Mice with gene deletion of CatK show impaired osteoclastic resorption which leads to osteopetrosis [6]. These mice display higher bone mass in both cortical and trabecular bone, greater trabecular and cortical thickness, and normal bone strength [7,8]. Mice overexpressing CatK show a decrease in trabecular bone volume of the distal femoral metaphyses and accelerated bone turnover [9]. Based on its substrate preference, cellular distribution, and genetic evidence, CatK is likely to play an important role in bone resorption. The selective inhibitor of CatK, odanacatib (ODN), is currently in development for the treatment of osteoporosis [10].

The preferred small animal model for evaluation of the efficacy of bone therapeutics is the ovariectomized (OVX) skeletally mature rat [11]. The utility of the OVX rat is limited by significant interspecies sequence variation (88% homology) in key residues within the active site of human and rat CatK enzymes [12]; inhibitors of human CatK have significantly lower potency against the rodent CatK enzyme to be reliably tested for bone effects in the rat or mouse model. In contrast, rabbit CatK shares 96% sequence identity and 99% similarity with its human counterpart with only two amino acid differences in the active site [12,13]. Therefore, the selection of rabbit as the preclinical species for *in vivo* screening assay for CatK inhibitors was due to the species differences in response to this class of bone resorption inhibitors. The differences in homologies between rat, rabbit and human CatK are derived from inhibitor potencies. For example, ODN is highly potent versus human and rabbit CatK (half maximal inhibitory concentration [IC_50_ ]= 0.2 and 1 nM, respectively) but more than 500-fold less potent versus rat CatK (IC_50_ = 112 nM) or mouse CatK (IC_50_ = 108 nM) [14]. Our work with the adult OVX rabbit shows that it is a relevant *in vivo* bone model for testing selective inhibitors of the human CatK enzyme [13]. However, the estrogen-deficient OVX model in skeletally mature rabbits requires approximately six months to achieve predictable and measurable bone loss by dual energy x-ray absorptiometry (DXA) [15]. Due to the long duration of the adult OVX rabbit model and the relatively large size of adult rabbits (3.5 kg), the use of the adult OVX rabbit model for quick *in vivo* screening and selection of compounds with limited drug quantity in the early pre-clinical phase is impractical.

A rapidly growing rabbit model has been developed for *in vivo* prioritization of CatK inhibitors before testing in the OVX rabbit assay. Development of this growing rabbit model is based upon the growing rat model used for testing the anti-resorptive efficacy of the bisphosphonates [16-18]. This model is often referred to as the “rat Schenk assay.” This assay relies upon inhibiting the process of bone resorption in rapidly growing animals at the periosteal surface (the “funnel region”) of the metaphysis, and the aspect of metaphyseal trabeculae in the marrow cavity that is opposite to the nearby epiphyseal growth cartilage. Inhibiting the removal of calcified cartilage by resorption in the primary spongiosa is also an important target. In early work, treatment of growing rats treated with the first-generation bisphosphonates, etidronate or clodronate, for ten days resulted in higher trabecular bone volume in the proximal tibial metaphysis of treated compared to untreated rats [16,17]. A more recent experiment showed increased trabecular bone volume following seven days of subcutaneous (SC) alendronate (ALN) (0.010 mg/kg/d) [18]. Furthermore, a more recent study using weanling rats showed an increase in distal femoral metaphyseal BMD following six weeks of once-weekly treatment with ALN [19]. A higher growth rate of the distal and proximal femur, the proximal tibia, and the proximal and distal radius has been observed in the rapidly growing rabbit than that typically observed with other species or in more mature animals [20,21].

The primary objective of the current study was to determine if the rapidly growing rabbit (“rabbit Schenk assay”) could be used to quantify and prioritize CatK inhibitors according to their respective potencies in inhibiting bone resorption *in vivo*. The model was first characterized by dose titrating ALN and determining areal BMD of the distal femur (DFBMD) using *ex vivo* DXA. Next, the efficacy of four relatively potent CatK inhibitors with varied chemical structures, *in vitro* potency, and *in vivo* pharmacokinetic profiles was assayed in a similar fashion.

## Methods

### Animals

Six- to seven-week-old female New Zealand White (NZW) rabbits (Covance Research Products, Denver, PA, USA), weighing approximately 1.35–1.5 kg were received. The rabbits were kept in wire-bottomed cages under standard laboratory conditions with lighting set for 12 h light, 12 h dark per 24 h, a constant temperature of 21 ± 3°C, relative humidity 50 ± 20%, and 10–15 air changes per hour. Rabbits received a standard pelleted diet (High Fiber Lab Rabbit Diet 5326, PMI Nutrition International, Brentwood, MO, USA), with water *ad libitum*. Animal experiments were reviewed and approved by the Institutional Animal Care and Use Committees at Merck Frosst Center for Therapeutic Research (Montreal, Quebec, Canada) and Merck Research Laboratories (West Point, PA, USA).

### Dose-ranging ALN in growing rabbits

To characterize the dose of ALN required to show increases in DFBMD, dose-ranging studies were conducted using ALN, administered at doses of 0, 50, 100, 200, and 500 μg/kg (SC, once daily). Seven-week-old rabbits were weight-randomized into groups (n = 8-11 per group). ALN was prepared in deionized water and pH adjusted to 7.2. The injection volume was 0.3 mL per rabbit. Rabbits were treated daily for 10 consecutive days. Rabbits were reweighed on Day 6 and dosing volume was adjusted accordingly. At necropsy, the right femur was excised and stored in 70% ethanol.

### CatK inhibitor screening

Rabbits were treated once daily by oral gavage for 10 consecutive days with vehicle (1% carboxymethylcellulose or 0.5%/0.2% carboxymethylcellulose/SDS), CatK inhibitors, or ALN (100 μg/kg, SC). Several studies were conducted, with each study including vehicle (n = 11–14), multiple dose levels of a CatK inhibitor (n = 13–14 per group), and ALN (n = 8–9). On Day 1, two rabbits from each group receiving CatK inhibitors were bled via the central ear artery (0.5 mL each) at 0, 0.25, 1, 3, 6, 8, and 24 h after dosing. Plasma concentrations of CatK inhibitors were determined in all samples. On Day 11, rabbits were euthanized. Femurs were removed and stored in 70% ethanol for BMD analysis.

### Enzyme inhibition assay (EIA)

Enzyme activity assays were carried out using rabbit CatK as previously described [22]. Briefly, the assay was carried out in 2-(*N*-morpholino) ethanesulfonic acid 50 mM pH 5.5 containing dithiothreitol 2.5 mM, ethylenediaminetetraacetic acid 2.5 mM, and 10% dimethyl sulfoxide. Prior to the addition of substrate, different concentrations of the inhibitor ranging from 100 μM to 0.2 nM were pre-incubated for 15 min with each enzyme (0.2–1 nM) to allow the formation of the enzyme-inhibitor complex. Substrate was then added and the enzyme activity measured from the increase of fluorescence at 460 nm 355 nm. The final volume of the reaction was 100 μL. Assays were performed in 96-well plate format and the plate was read using a Spectramax (Molecular Devices) plate reader. The percentage inhibition of the reaction was calculated from a control reaction containing only vehicle. IC_50_ curves were generated by fitting percentage inhibition values to a four-parameter logistic model (SOFTMAX PRO, Molecular Devices, Sunnyvale, CA, USA).

### Bone resorption assay

The bone resorption assay is a functional *in vitro* assay that measures Type-I collagen degradation after a three-day incubation of rabbit osteoclasts cultured on bovine bone with varying concentrations of test compound, as previously described [23]. Briefly, long bones isolated from a 10-day-old NZW rabbit were finely minced in alpha-minimal essential medium (α-MEM) (Gibco BRL; Gaithersburg, MD, USA) containing penicillin/streptomycin, pH 7.1 to obtain a cell suspension and 1 × 10^6^ cells were seeded onto each 6 mm diameter × 0.22 mm thick bovine bone slice in the same medium containing 2% fetal bovine serum (FBS). After 4 h, the medium was replaced with α-MEM, 2% FBS, 1,25(OH)_2_D_3_ 10 nM, and test compounds. The cultures were incubated for three days at 37°C in 5% CO_2_. C-telopeptide of Type-I collagen (CTx-I) released into the medium was measured by the CROSSLAPS Elisa assay (Osteometer Biotech, Herlev, Denmark).

### BMD analysis

Whole right femurs with muscles removed, were immersed in two inches of water in an acrylic box, and positioned with both distal condyles resting on the bottom of the box. The distal 5 cm of the femur was scanned using small animal software in high resolution mode on a Hologic QDR 4500 fan-beam bone densitometer (DXA; Hologic, Inc., Waltham, MA, USA). The distal 3 cm of the femur was analyzed. A region of interest (ROI) beginning one line distal to the distal edge of the femur and centered 70 lines wide and extending 60 lines proximally in the long axis of the bone was applied. Bone mineral content (BMC) and bone area (BAr) were output by DXA software. BMD was calculated as BMC/BAr.

### Histological examination of distal femur

Following *ex vivo* DXA scanning, the distal one-third of the femur was cut mid-sagitally and then dehydrated, without prior decalcification, in increasing concentrations of ethanol. The right portion was embedded in 80% methylmethacrylate/20% dibutyl phthalate. Parasagittal sections (6 μm) were cut on a Reichert-Jung Polycut sledge microtome (Nussloch, Germany) and mounted on glass slides. A Masson’s trichrome stain was performed to view calcified tissue.

### Statistical analysis

For dose-ranging ALN and CatK inhibitor L-833905 studies, DFBMD differences of treatment groups compared to vehicle were analyzed by Kruskal-Wallis non-parametric analysis of variance (ANOVA) with Student-Neuman-Keuls *post hoc* testing. Differences were considered significant when p ≤ 0.05. All comparisons were made using CRUNCH software (JanDel Corp.; San Jose, CA, USA). For all other studies of CatK inhibitors, statistical computation of DFBMD data was performed using Statview (SAS Institute, Inc., Cary, NC). Differences among treatment groups were tested by one-way ANOVA. If significant differences were indicated by ANOVA, comparison between group means was tested by Fisher’s partial least-squares difference for *post hoc* analysis. Differences were considered significant when p ≤ 0.05.

## Results

### Characterization of the rabbit Schenk model in growing rabbits

Early characterization of the rabbit Schenk model included dose titration of ALN and suitable CatK inhibitors developed early in the screening program. Experiments using ALN at 50, 100, 200, or 500 μg/kg/day SC for ten consecutive days showed significant increases in DFBMD above vehicle, ranging from 15% at 50 μg/kg/day (p < 0.01) to 21% at 500 μg/kg/day (p < 0.001) (Table 1).

**Table 1:** Effect of 10 days' ALN treatment on DFBMD in growing rabbits

**ALN dose (μg/kg)**	**DFBMD (mg/cm**^**2**^**)**		**% BMD gain**
	**Veh**	**ALN**	
50	241.4 ± 9.2	277.8 ± 8.4**	15
100	270.5 ± 7.9	313.7 ± 3.9***	16
200	242.0 ± 3.4	281.7 ± 6.4***	16
500	241.6 ± 5.2	291.2 ± 9.4***	21

Mean ± standard error of the mean; ^**^p < 0.01, ^***^p < 0.001 vs. vehicle.

ALN = alendronate, DFBMD = distal femoral bone mineral density, Veh = vehicle.

Importantly, these results, that tested doses of ALN that varied by an order of magnitude, established the “rabbit Schenk assay” as one that could assess anti-resorptive efficacy, by testing an established osteoporosis treatment agent that had been previously tested in the rat Schenk assay [18]. ALN 100 μg/kg/day was selected as a reliable positive control for subsequent studies that tested CatK inhibitors in growing rabbits.

**Figure 1:**
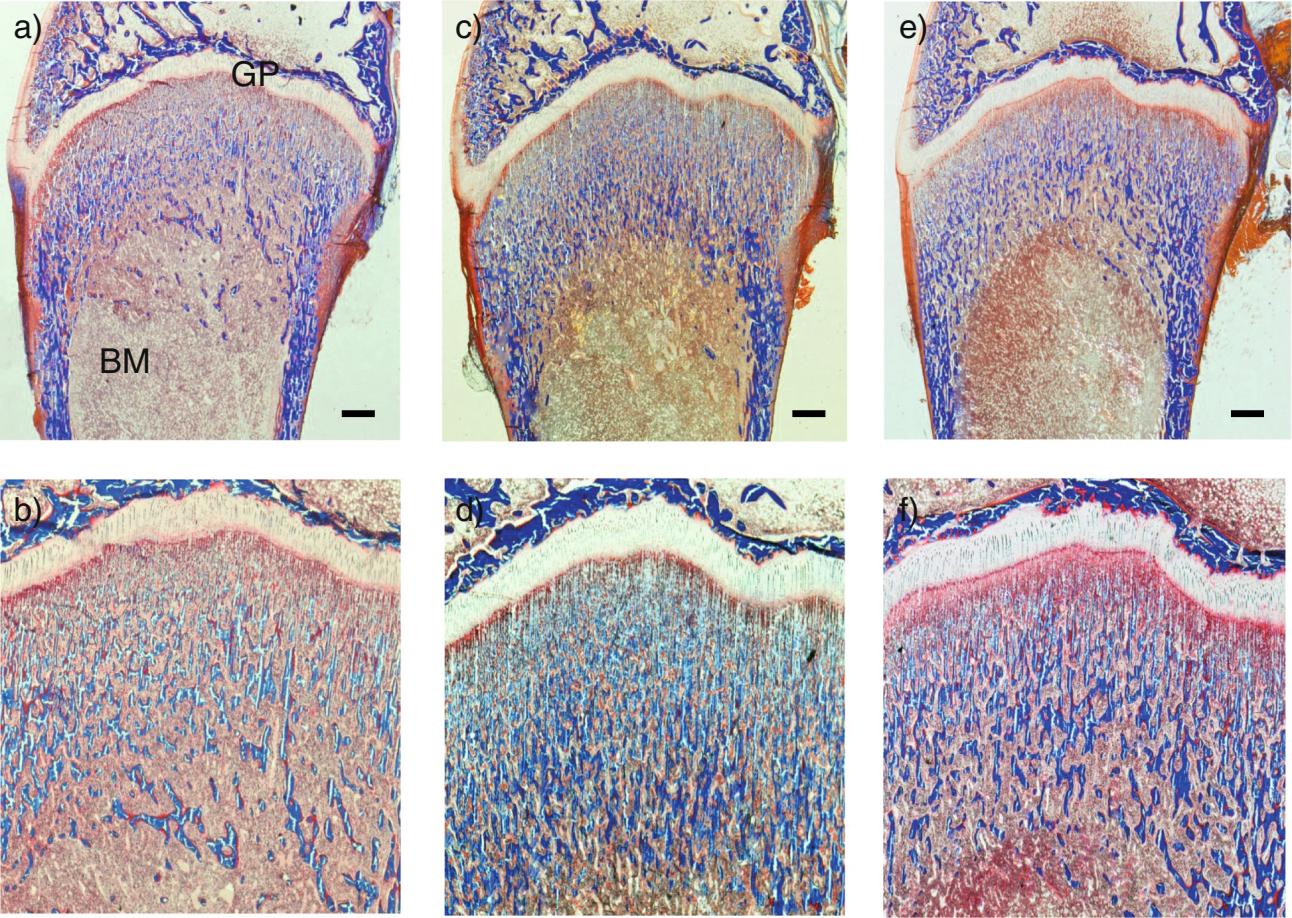
**Increased retention of calcified cartilage and bone in response to treatment with CatK inhibitor or ALN.** Representative histological images of distal femoral region from rabbits treated with **(a, b)** vehicle, **(c, d)** alendronate, and **(e, f)** a cathepsin K inhibitor for 10 days are shown. Distal femurs **(a, c and e)** and their metaphysis regions **(b, d and f)** are shown at 0.5X and 1X, respectively. Bone, blue trabecular spicules; BM, bone marrow; GP, growth plate. Scale bar = 1 mm.

We also histologically examined the treatment-related effects of bone resorption inhibitors, including both ALN and a CatK inhibitor that was selected as a tool compound from an early chemical series of the screening program (Figure 1). The metaphyseal region of a rapidly growing rabbit showed high levels of modeling associated with calcified cartilage septae and trabecular bone (Figure 1a&b). Treatment with either ALN (Figure 1c&d) or a CatK inhibitor (Figure 1e&f) resulted in increased retention of both calcified cartilage and bone tissue, when compared to vehicle treatment. No obvious morphological changes in the growth plate, cortical bone and bone marrow were noted in animals treated with these bone resorption inhibitors for 10 days.

### Evaluation of *in vivo* efficacy of potent CatK inhibitors

#### Selection of CatK inhibitors based on in vitro potencies

We highlight here four CatK inhibitors selected for validation in the rabbit Schenk assay, based on their *in vitro* profiles. Table 2 summarizes the IC_50_ values of the CatK inhibitors: L-833905 ((R)-N-(cyanomethyl)-4-methyl-2-(4′-(piperazin-1-yl)-[1,1′-biphenyl]-3-yl)pentanamide) [24], L-006235 [13], L-873724 [25] and L-1037536 (ODN) [25] tested against rabbit CatK using 2 μM of Z-Leu-Arg-AMC as substrate [13,25]. IC_50_ values for the same compounds in the functional bone resorption assay are also shown in Table 2[13,25]. In the functional bone resorption assay, L-006235, L-873724, and ODN were potent inhibitors of bone resorption *in vitro*. These three compounds were approximately 30-fold more potent in the bone resorption assay than L-833905 [13,25]. Though potencies obtained in the bone resorption assay were reduced by an approximate 5–20-fold shift relative to those in the enzymatic assay, the same rank order of potencies was maintained.

**Table 2:** **Pharmacokinetic profiles of CatK inhibitors in rabbits**[13,25]

**Compound**	**Dose (mg/kg)**	AUC (μm*hr)	**C**_**max **_**(nM)**	**C**_**min **_**(nM)**	**Cl (mL/min/kg)**	**[C**_**min**_**]/bone res IC**_**50**_	**IC**_**50 **_**(nM)**	
							**Rabbit CatK**	**Rabbit bone resorption assay**
L-833905	10	1.9	163	29	–	0.2	33 ± 2	149 ± 20
	30	6.1	651	60		0.4		
L-006235	1	0.3	74	9	113	1.8	0.5	5 ± 1
	3	1.0	337	15		3.0		
	10	5.0	2436	8		1.6		
L-873724	1	0.5	78	<LOD*	26	≤0.1	0.8	13 ± 1
	3	3.1	490	28		2.2		
	10	7.8	1370	32		2.5		
ODN	0.3	0.7	33	35	6	1.5	1.0	23 ± 6
	1	2.2	130	37		1.6		
	3	4.1	270	88		3.8		

* ~ 1 nM.

AUC = area under the curve, CatK = Cathepsin K, Cl = clearance, C_max_ = maximum concentration after dosing, C_min_ = minimum concentration after dosing, IC_50_ = half maximal inhibitory concentration, LOD = limit of detection, ODN = odanacatib, res = resorption.

Reprinted (adapted) with permission from Falgueyret et al. 2005 [13].

Reprinted (adapted) with permission from Gauthier et al. 2008 [25].

#### Pharmacokinetic evaluation of CatK inhibitors in growing rabbits

Doses of the respective CatK inhibitors for the rabbit Schenk assays were selected on the basis of trough plasma levels (C_min_) following oral dosing in the same species. As indicated in Table 2, the mid-dose of all inhibitors, except L-833905, provided trough plasma levels that remained at or above the rabbit bone resorption IC_50_ for the duration of the study (i.e. C_min_ at 24 h following oral dose ≥ bone resorption IC_50_ or [C_min_]/bone resorption IC_50_ ≥ 1). For both doses of L-833905 and the lowest dose of L-873724, the plasma levels dropped below the bone resorption IC_50_ at 24 h (Table 2). Although ODN was slightly less potent compared to L-006235 and L-873724 in the bone resorption assay, lower doses were selected due to its longer rabbit plasma half-life which thereby provided higher C_min_ plasma levels at 24 h post-dosing.

#### Effects of CatK inhibitors on distal femoral BMD (DFBMD)

**Figure 2:**
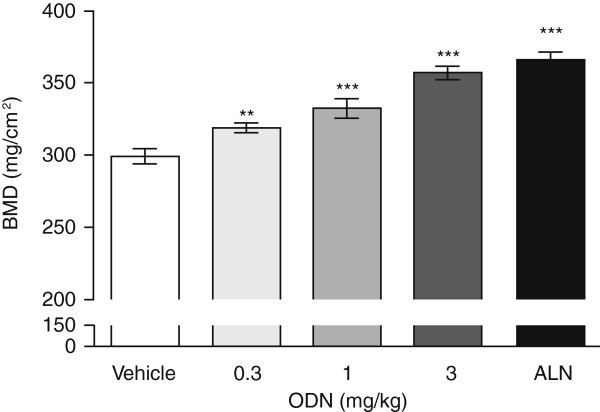
**Relationship between treatment with ODN or ALN and DFBMD.** Treatment with ODN or ALN increased DFBMD in the growing rabbit model. Mean ± standard error of the mean; ALN = alendronate, DFBMD = distal femur bone mineral density, ODN = odanacatib.

**Figure 3:**
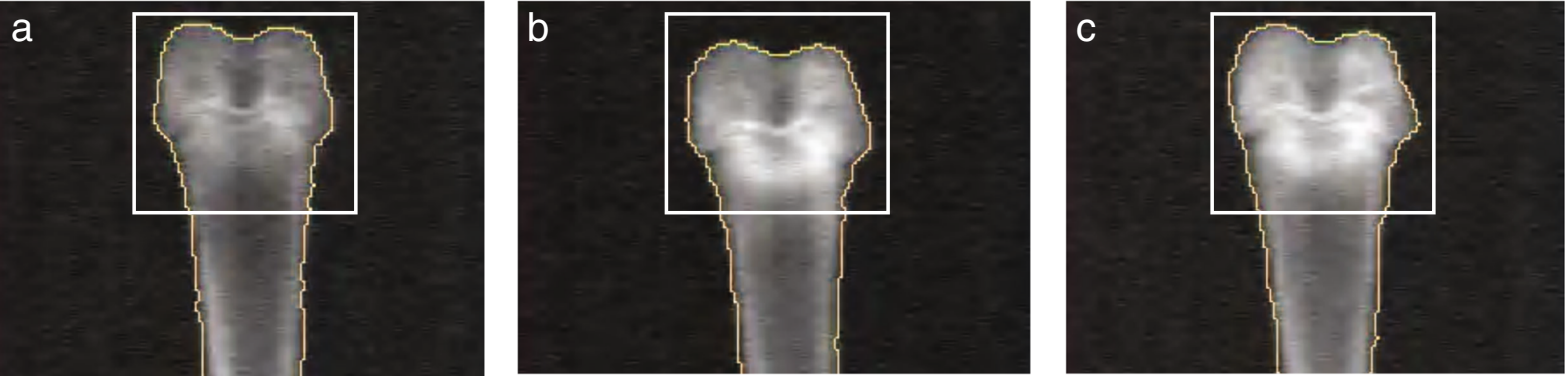
**Representative DXA images of the rabbit distal femur after 10 days' treatment with ALN or ODN.** Note increased density of secondary spongiosa with ALN or ODN as compared to vehicle. **(a)** vehicle (DFBMD = 263 mg/cm^2^), **(b)** ALN (DFBMD = 361 mg/cm^2^), and **(c)** ODN 3 mg/kg (DFBMD = 388 mg/cm^2^). ALN = alendronate (100 μg/kg/day), DFBMD = distal femur bone mineral density, DXA = dual energy x-ray absorptiometry, ODN = odanacatib.

Table 3 shows DFBMD values in response to dosing with three CatK inhibitors. Rabbits treated with ALN had significantly higher DFBMD than vehicle in each experiment. DFBMD with L-833905 was numerically higher at 10 mg/kg (3%, NS) and significantly higher at 30 mg/kg (10%, p < 0.01). Treatment with 1 mg/kg L-006235 resulted in a statistically significant increase in DFBMD (7%, p < 0.01), while the 3 and 10 mg/kg groups showed robust increases in DFBMD (11% and 15%, respectively, both p < 0.001). Rabbits treated with L-873724 had higher DFBMD at 3 (9%, p < 0.01) and 10 mg/kg (15%, p < 0.001) than vehicle. ODN treated rabbits had higher DFBMD, that was significantly greater than vehicle at 0.3 (7%, p < 0.01), 1 (11%, p < 0.001), and 3 mg/kg (19%, p < 0.001) (Figure 2). Figure 3 highlights the increases in DFBMD by both ALN and ODN compared to vehicle control.

**Table 3:** **Distal femur BMD (mg/cm**^**2**^**) in three rabbit Schenk assays**

**Compound**	**Vehicle**	**Dose**				**ALN**
		**1 mg/kg**	**3 mg/kg**	**10 mg/kg**	**30 mg/kg**	
L-833905	274.3 ± 4.5	–	–	282.5 ± 5.5	301.8 ± 4.9^**^	314.0 ± 9.1^**^
L-006235	263.3 ± 4.5	281.7 ± 4.9^**^	293.5 ± 2.8^***^	303.5 ± 5.4^***^	–	300.5 ± 4.6^***^
L-873724	285.5 ± 5.4	301.2 ± 3.7	310.5 ± 6.5^**^	327.7 ± 7.5^***^	–	352.8 ± 9.4^***^

Mean ± standard error of the mean; ^**^p < 0.01, ^***^p < 0.001 vs. vehicle.

ALN = alendronate, BMD = bone mineral density.

## Discussion

CatK is predominantly expressed in osteoclasts [1], and has an important role in the degradation of the collagen matrix components of bone (predominantly Type-I collagen) at acidic pH. Based on human genetics [3-5], experimental genetics in mice [6], substrate preference, and cellular distribution, the pivotal role of CatK in osteoclastic bone resorption has been demonstrated. These findings have led to the development of pharmacologic inhibitors of CatK to treat diseases characterized by high bone turnover such as osteoporosis.

In this report, four CatK inhibitors, L-833905, L-006235, L-873724, and ODN, were evaluated for their effects on DFBMD in the rapidly growing rabbit. Based on their respective pharmacokinetic profiles in rabbits and their *in vitro* profiles, these compounds were chosen to evaluate *in vivo* anti-resorptive efficacy. These four compounds, when administered daily for 10 consecutive days to growing rabbits, significantly increased DFBMD versus vehicle treatment in a dose-dependent fashion, showing that inhibitors of the CatK enzyme inhibit bone resorption *in vivo*, as predicted by their respective potencies in the EIA and the *in vitro* bone resorption assay, and their pharmacokinetic profile in rabbits.

From the EIA and functional cell-based assay, L-006235, L-873724, and ODN were selected as potent inhibitors of CatK-mediated activity of rabbit osteoclasts in the degradation of Type-I collagen *in vitro.* L-833905 was a 6–30-fold less potent inhibitor of CatK than the above three compounds. The rank order of potencies of these inhibitors in the bone resorption assay tracked well with that in the EIA. The shift in potencies between the two assays may reflect the degree of protein binding of the inhibitors and high fractional inhibition of CatK required to inhibit bone resorption in osteoclasts [13,25]. Moreover, the potencies of these compounds were also dependent on their ability to penetrate and exit the resorption lacunae and lysosomes of osteoclasts during resorption [26].

Based on our historical database, inhibitors of the human CatK enzyme are about two orders of magnitude less active in inhibition versus the rat CatK enzyme. The development of a potent and selective inhibitor of rat CatK versus other rat cathepsins has been shown to be challenging [27,28]. However, human CatK inhibitors are generally effective in the rabbit, usually displaying only approximately 5-fold less potency toward the rabbit enzyme. These findings are supported by the higher amino acid sequence homology between human and rabbit enzymes. These fundamental interspecies differences in CatK led us to select the growing rabbit as a relatively small size animal model, which can be used to rapidly identify *in vivo* anti-resorptive activity of numerous CatK inhibitors prior to the evaluation of selected candidates in the long-term OVX model of postmenopausal osteoporosis in the adult rabbit or non-human primate (NHP).

The growing rabbit has several advantages, including a higher bone growth rate than that typically observed with other laboratory large species such as dog, pig or monkey, which have been used previously to evaluate therapeutic agents for osteoporosis [20,21]. We previously described the development of the adult OVX rabbit assay to assess the efficacy of CatK inhibitors on preventing estrogen deficiency bone loss in the lumbar vertebrae and distal femur [15]. However, the adult OVX rabbit model has many limitations which preclude its use for routine drug screening, including limited availability of skeletally mature aged rabbits, requirements for surgical manipulation, long study duration and a large body weight that requires preparation of large quantities of agents. An NHP *in vivo* screening assay examining markers of bone resorption, (e.g. serum CTx, collagen Type-I N-telopeptides) [27,29] also has disadvantages, including availability of trained personnel, long washout periods, high demand for drug quantity, high cost, and limited numbers of skeletally mature NHPs available for drug screening purposes.

Thus, a short-term reliable *in vivo* screening assay to quickly assess potencies of compounds for further optimization was highly valuable for a drug screening program. The current study is the first to report the use of the rapidly growing rabbit as an assay for *in vivo* activity of bone resorption inhibitors, with similar fundamental characteristics to that previously used in growing rats [16-18,30,31]. Unlike the adult OVX rabbit, this model requires only 10 days of dosing. Considering that the assay itself is 18 times shorter in duration and the weight of the animals during the assay is 40% of those used in the adult OVX rabbit model, the total requirement for compound is 40-fold less than that needed for an adult OVX rabbit study. Efficacy of the bone resorption inhibitors is assessed by *ex vivo* DXA of the distal femur, a region that contains an active epiphyseal growth cartilage in growing rabbits. During longitudinal growth, the structure and density of metaphyseal trabecular bone relies on a well-controlled balance between calcified cartilage formation in the zone of cell hypertrophy of the epiphyseal growth cartilage, bone deposition in the primary and secondary spongiosa, and the removal of both calcified cartilage and bone in both the primary and secondary spongiosa. Inhibition of calcified tissue resorption during growth without effects on chondrocyte activity in the epiphyseal growth cartilage results in a density increase in the primary and secondary spongiosa that is characterized by higher trabecular number. The higher trabecular number is due to an increase in the number of persisting calcified cartilage septa upon which new bone tissue is deposited. Bisphosphonates increase metaphyseal trabecular bone volume and trabecular number in the proximal tibial metaphysis of the growing rat [16-18,30,31]. In addition, when non-decalcified histologic sections are used, the rat Schenk assay becomes useful for screening for the existence of mineralization defects [16,17].

The results of these rabbit Schenk studies suggest that potent and orally active CatK inhibitors are effective as bone resorption inhibitors *in vivo*. This assay can be used to quickly rank order potencies of the CatK inhibitors prior to their evaluation in estrogen deficiency-related bone-loss studies. In fact, efficacy of the CatK inhibitors L-006235 and ODN were further demonstrated in adult OVX rabbits [15] and ODN in OVX NHPs [32,33].

## Conclusions

The rabbit Schenk assay is a valid, consistent *in vivo* screen for two classes of bone resorption inhibitors, CatK inhibitors and bisphosphonates. The current data confirm the efficacy of four different selective CatK inhibitors and ALN in reducing bone resorption *in vivo* in the rapidly growing rabbits. The results for CatK inhibitors were well-correlated with those in the *in vitro* bone resorption assay. The Schenk assay results testing L-006235 and odanacatib predict the outcome of tests of the same agents on estrogen deficiency-induced bone loss in both skeletally mature rabbit [14] and monkey models [32]. Therefore, while the OVX animal model is the standard method for assessing efficacy and long-term effects of anti-osteoporosis agents on bone quality, we demonstrated that the rabbit Schenk assay can serve as a rapid, cost-effective, and reliable test for evaluation of numerous bone resorption inhibitors prior to the evaluation of a few selected candidates in the long-term OVX-model of the same species or non-human primates.

## Abbreviations

ALN: Alendronate; ANOVA: Analysis of variance; AUC: Area under the curve; BAr: Bone area; BMC: Bone mineral content; BMD: Bone mineral density; CatK: Cathepsin K; Cl: Clearance; Cmax: Maximum concentration after dosing (peak plasma levels); Cmin: Minimum concentration after dosing (trough plasma levels); CTx-I: C-telopeptide of Type-I collagen; DFBMD: Distal femur bone mineral density; DXA: Dual energy x-ray absorptiometry; EIA: Enzyme inhibition assay; FBS: Fetal bovine serum; IC50: Half maximal inhibitory concentration; LOD: Limit of detection; α-MEM: Alpha-minimal essential medium; NHP: Non-human primate; NZW: New Zealand White; ODN: Odanacatib; OVX: Ovariectomized; ROI: Region of interest; SC: Subcutaneous; SDS: Sodium dodecyl sulfate.

## Competing interests

All authors were Merck’s employees during the execution of the studies as disclosed in this manuscript, and may own stock or stock options in the company. DK has received personal fees from Amgen, Bayer, Lexicon, Xradia and Arcarios.

## Authors’ contributions

Study design: DK, RO; Assisting with study design: SL; Performing the experimental work: SL, BP; Performing the statistical analysis: DK, BP; Drafting of the manuscript: BP, RO, DK, LD, SL; All authors have read and approved the final manuscript.

## Pre-publication history

The pre-publication history for this paper can be accessed here:

http://www.biomedcentral.com/1471-2474/14/344/prepub
